# Błażek Ambivalent Parental Communication Questionnaire (BAPCQ)

**DOI:** 10.3390/ijerph20064987

**Published:** 2023-03-12

**Authors:** Magdalena Błażek, Natalia Nadrowska

**Affiliations:** Department of Quality of Life Research, Faculty of Health Sciences, Medical University of Gdansk, 80-210 Gdansk, Poland

**Keywords:** consistency, clarity, ambivalent communication, development, security, health

## Abstract

Communication is one of the three overarching processes of family resilience, along with the belief system and organizational processes of family life. Honest, unambiguous communication with a child is one of the important bases of a child’s development and feeling of security and healthy functioning in relations. The aim of our research was to construct a questionnaire aimed to measure consistency in communication on two dimensions: verbal and non-verbal communication, statements, and actions of parents. In this study, 404 persons participated: 319 (79.0%) women and 85 (21.0%) men, aged 18 to 61 (M = 24.83, SD = 7.87). Confirmatory factor analysis confirmed a two-factor model with 52 items that were well-fitted to the data for both versions. The model indicators were found to be well suited to the data (for communication with the mother were: χ2 /df = 1.58, RMSEA = 0.03, CFI = 0.99, TLI = 0.99, SRMR = 0.06, and for the communication with father version: χ2 /df = 2.34, RMSEA = 0.05, CFI = 0.98, TLI = 0.98, SRMR = 0.07). The Błażek Ambivalent Parental Communication Questionnaire (BAPCQ) could be used in a clinical context as well as in scientific studies and is designed to test adults who evaluate their communication with their parents.

## 1. Introduction

Both, general communication models and those specifically dedicated to human functioning within the family, emphasize the importance of clear, open communication at the emotional and informational levels. It allows not only for more effective action, but is also one of the key factors in building strong, stable social/family bonds. According to the Family Communication Patterns theory developed by Koerner and Fitzpatrick [1] members of families share schemas of communication because they need accuracy and congruence, and this need could be satisfied by developing through communication and shared social reality. The main purpose of developing the questionnaire presented in this article was the analysis of family communication from the perspective of an adult child. We wanted to measure the ambivalence in communication not only from the diagnostic, i.e., how it affects the quality of a person’s functioning but also from the potential therapeutic perspective. Communication patterns in terms of consistency vs. ambivalence have a significant impact on the lives of all family members, and for children, it influences the way of building and maintaining relationships in adult life. Those patterns can be worked on with families in order to improve them and make them more effective both from the perspective of clarity at the emotional level, as well as in building orientation regarding trust, expectations, and approved course of action. Although there are several methods developed to asses family communication (see Table 1), we believe that our questionnaire offers a more detailed assessment of what aspects of communication are a resource and which are problematic in terms of consistency and ambiguity.

Family resilience enables a family to withstand a difficult, overwhelming time, helps to cope with a crisis, and gives the opportunity to emerge from the crisis as empowered and resourceful. Stressors in terms of family resilience are understood as challenges facing the family [2,3]. Challenges foster the development potential of the whole family and its members. Effective overcoming and coping with everyday stress, major or minor difficulties, and normative and non-normative crises is a resource for the family that can be used in future stages of family life [4,5].

Communication is one of the three overarching processes of family resilience, along with the belief system and organizational processes of family life [6]. These processes are synergistic and interactive, and cooperate in various areas of family life. Each of these processes is unique and used by the family depending on previous crisis events, the stage of family life, the specificity of the stressor, and the family’s reaction to it, as well as available coping strategies and support networks in the extended family or community. Each of these three over-arching processes consists of three sub-processes. For the family communication process, these are clarity of communication, open emotional sharing, and collaborative problem-solving. The organizational process of family life consists of flexibility, connectedness, and mobilizing social and economic resources, while the belief system includes making meaning of adversity meanings, positive outlook, transcendence, and spirituality [7].

The sharing of truthful, consistent, and unambiguous information by family members is a process of communication clarity. In line with the assumption of this process, the family seeks to search for knowledge related only to the current situation, and ignore redundant or unnecessary information [8,9]. Clarifying ambiguous situations enables the family to achieve peace and inner balance and to feel secure when facing life challenges and stress. Sharing information with other family members, without keeping crucial matters hidden, can contribute to understanding the causes of a difficult situation and seeing opportunities to resolve the crisis. Clarity of communication is also important to the process of open emotional expression [10]. Sharing positive and negative feelings instead of experiencing them alone, and tolerance towards the different feelings of other family members instead of forbidding them reveals them, helps to build a climate of mutual respect. Similarly, clarity of communication is essential in the collaborative problem-solving process [11,12,13,14]. Acceptance of crisis by the family, preparing an action plan, and taking steps to solve a stressful situation that the family faces is only possible with clear and unambiguous communications [15]. By expressing themselves in a clear, understandable, and authentic way, family members create an area for growth for positive relationships based on honesty, shared responsibility, respect, and cooperation [16]. Communication patterns in the family, which condition the maintenance of an appropriate level of family cohesion and flexibility, are conducive to coping with adversities [17,18]. There is a significant relationship between early childhood negative and harmful experiences and the health condition in adulthood [19]. Ambivalent and incoherent communication in the family is one of the risk factors for the emergence of psychological problems both in the emotional sphere (e.g., depressive states) and in the cognitive sphere (e.g., dysfunctional cognitive schemas). As a consequence, such a person experiences difficulties in building lasting and satisfying relationships with others, which in turn impairs his or her overall functioning [20]. On the other hand, proper communication in the family is a protective factor that allows the development of high personal competences related to emotional regulation and social life [21]. It can therefore be said that even in families experiencing crises, clear, open communication will be a factor protecting against the long-term consequences in the form of problems conditioned by emotional dysfunctions.

The reasons for ambivalent communication behavior can be found both in specific situations and in communication between people in their families of origin [22,23]. With regard to the first aspect, unclear communication is understood as ambiguous situations that do not offer one correct answer, as well as situations in which there is a lack of information that would help clarify an event [24,25]. Examples of such situations include: a family member missing in unknown circumstances, the disappearance of a loved one without a body to be buried, or dementia, when we are in a relationship with a loved one despite mental separation. With regard to ambiguous loss, resilience will help in dealing with long-term uncertainty and tolerating ambiguity [26,27]. Referring to specific family relationships, the basis for explaining contradictory communication, also called paradoxical, may be the concept of double bind presented by Bateson [28]. This theory involves a conflict that cannot be solved when the child depends on the caregiver. The parents’ statements contradict each other, thus creating confusion and internal chaos in the child. If a child were to point out to a parent that there was a lack of clarity in his or her communication, it would result in the caretaker’s disapproval and the loss of his or her love [29,30].

Communication is a series of complex, intentional, and consecutive processes containing a multitude of information. Therefore, the processes of clarity of communication and emotional openness and cooperation in solving problems can be expressed in at least two levels of communication in terms of consistency-ambivalence. First, in what the person says in relation to what they do. Second, with regard to verbal versus non-verbal communication [31]. 

Compatibility-incompatibility at the level of spoken words and actions largely refers to promises made, trust in others, the contradiction of one’s own words and fulfilment of one’s obligations. In terms of promises, it expresses the discrepancy between making and delivering on promises that cannot be made, empty promises, and denying one’s previous words. Trust means being able to rely on loved ones, not breaking promises or failing someone by not fulfilling expectations [32]. Acting in accordance with proclaimed views, fulfilling one’s duties, inconsistency in one’s own actions, the effective realization of plans, giving up activities already in progress, and forgetting about obligations are covered by the concept of contradicting one’s words and fulfilling one’s obligations [29].

Two aspects of verbal-non-verbal communication can be highlighted: perceived verbal and non-verbal messages, and compliance with the real thoughts and feelings of a given person, as well as perceived verbal and non-verbal messages with regard to family relationships. In the first aspect, there are problems with understanding the real intentions and feelings of the converser, and in the second, through inconsistency, the person tries to control the relationship, alternately distancing and becoming closer [33].

An important aspect of research on the family or therapeutic work with the family is to recognize the shortcomings and needs of the family in the area of adequate and clear communication. We have reviewed the methods in terms of family communication, and these methods only briefly focus on the clarity of the message and the perceived ambivalence in it. Our review of methods includes questionnaires such as Family Resiliency Assessment Questionnaire [34], Walsh Family Resilience Questionnaire [23], Family Assessment Scales [35], Marriage Communication Questionnaire [36], Family Relations Questionnaire [37], Ambivalence over Emotional Expression Questionnaire [13], Family Expressiveness Questionnaire [38], Communication patterns questionnaire [39]. These questionnaires examined many aspects of communication, but none of them strictly dealt with discrepancies in the different levels of communication. The subscales of questionnaires measuring family communication are presented in Table 1.

We would like to emphasize that the lack of clear and consistent communication makes it difficult to adjust and adapt to constantly changing living conditions. No matter what type of communication incompatibility a family faces, it always creates confusion and introduces unnecessary chaos to the reality in which the family lives. The resilient process of communication and problem-solving enables changes to be made in the process of family beliefs or the process of organizing family life. Effective communication is the ability to manage a family in stressful crisis situations [40]. By communicating with each other, family members acquire a clear image of the situation in which they are found. It is reasonable to notice the discrepancies appearing in communication and attempt to reveal them, which will help improve communication between family members in the future.

## 2. Materials and Methods

### 2.1. Aims of the Study

The study aimed to develop a method for retrospective measurement of the consistency between verbal and non-verbal communication, as well as statements and actions of parents as assessed by their adult children. We decided to create two versions of the same questionnaire. In the first version, the respondents assessed their communication with their mother, and in the second—with their father. 

### 2.2. Ethics

We obtained permission from the Bioethics Committee for Scientific Research at the Medical University of Gdańsk (protocol code NKBBN/325/2021 and date of approval: 8 April 2021), Poland. Informed consent was collected from all participants.

### 2.3. Participants

We recruited 404 participants: 319 (79.0%) women and 85 (21.0%) men, aged 18 to 61 (M = 24.83, SD = 7.87). The largest group was people with secondary education (*n* = 279, 69.1%), living in cities with more than 500,000 inhabitants (*n* = 195, 48.3%). the characteristics of the group are presented in Table 2.

### 2.4. Questionnaire Development

Work on the Błażek Ambivalent Parental Communication Questionnaire (BAPCQ) began with the description preparation of two scales: statement and action consistency, and verbal and non-verbal consistency. The scales of the questionnaire were based on several models: autotelic family context developed by Csikszentmihalyi [41], Olson’s Circumplex Model of Marital and Family Systems [42], and Bateson’s [28] double bind model. The concept was also based on Błażek’s 25 years of psychological experience with families in normative and non-normative crises [33]. We put forward a thesis that the consistency of communication in compliance between declarations and actions as well as verbal and non-verbal communication is quite universal, at least in Western culture. It is also a very important protective factor. Firstly, it protects against the appearance of psychological problems resulting from the unpredictability of the world, and secondly, it creates conditions for the full development and use of a child’s individual potential. Our work on the questionnaire was also based on the ecological model of psychological assessment which assumes that questionnaires designed to examine various aspects of psychological functioning should contain questions that are understandable in terms of describing the real-life experiences of people.

The next step involved students of psychology generating items corresponding to these characteristics (*n* = 40). We obtained a list of 200 items, which were then linguistically analyzed by a language expert. A total of 83 unclear, repetitive and semantically similar items were removed. Then two experts in the field of family psychology received a detailed description of the scales based on which they assessed the compliance of each item in the questionnaire on a 5-point scale (from 1—completely not to 5 completely yes). We decided to leave only the items rated as completely yes. The final version of the questionnaire consisted of 62 items and was prepared separately for the assessment of communication with the father and mother. The statement-action consistency scale consisted of 27 items, and the verbal-non-verbal consistency consisted of 35 items. The following stage of the study was for the target group to fill out the questionnaire. Links to the questionnaire were posted on family life websites. The inclusion criterion was: age over 18 and brought up by parents (mother or father or both). Participants rated each item on a 5-point scale, from 1 (never) to 5 (always). 

### 2.5. Validation Methods

Three scales were used to determine the validity criterion of the BAPCQ. The first scale was the Family Communication and Problem Solving (FCPS) from the Family Resilience Assessment Scale PL (FRAS-PL) questionnaire [43] and this scale refers to sharing of information and feelings in a clear and open way when solving problems. The scale consists of 27 statements and the respondents assess the truthfulness of the items on a 4-point scale from 1 (strongly disagree) to 4 (strongly agree). 

The second scale was the Communication Process from the Walsh Family Resilience Questionnaire PL (WFRQ-PL) [44]. This scale relates to clear family communication, open emotional expression, and cooperation of family members in solving problems. Respondents rate 10 items on a 5-point scale from 1 (rarely/never) to 5 (almost always).

The third scale was Family Communication from the Family Assessment Scale (SOR) Questionnaire [35]. This scale focuses on the ability of the family or partner system to communicate with each other in a correct way. Family members on a 5-point scale from 1 (completely disagree) to 5 (fully agree) assess 10 items.

### 2.6. Statistical Analysis

After collecting a sufficient number of participants, we proceeded to statistical analysis. We performed a confirmatory factor analysis with the DWLS estimator to obtain an appropriate fit of the model with the data, by using the following indicators: RMSEA, CFI, TLI, and SRMR. Next, we removed items with low factor loading (<0.30). We decided to maintain the equivalence of items in both versions of the questionnaire so we removed items whose value was correct in one version but low in the other one. Then we replayed CFA to obtain a better model fit and used Cronbach’s alpha to determine the reliability of the final version of the questionnaire and Pearson’s correlation coefficient to determine criterion validity. We also conducted Harman’s one-factor test for common method bias for both versions of the questionnaire. In the data, we obtained there is no problem with common method bias since the total variance extracted by one factor is 43.16% for the version of communication with the mother and 44.53% for the version of communication with the father and it is less than the recommended threshold of 50%. We performed statistical analyses with the use of R v. 4.0.5, RStudio v. 1.4.1717, and SPSS v. 27 software.

## 3. Results

### 3.1. Confirmatory Factor Analysis

We performed confirmatory factor analysis three times to obtain the best-fit model to the data for two versions of the same questionnaires (for communication with the mother and for the father separately). The results of subsequent confirmatory factor analyses for them are presented in Table 3, whereas the steps that allowed us to achieve the final result are described below. 

Firstly, we tested the model with 62 items, 27 for the verbal-non-verbal consistency scale and 35 for the statement-action consistency scale. This analysis allowed us to select questions with low factor loadings (<0.30). There were eight items in the communication-with-mother version: 33, 41, 46, 48, 50, 52, 57, 59, and seven items in communication-with-father version: 18, 38, 41, 46, 48, 50, 59. Table 4 shows the load values for the 62-item model.

Secondly, we examined the two-factor model with 54 items for communication with the mother and with 55 items for communication with the father. We carried out the confirmatory factor analysis again, not including data from items with low factor loadings. The models turned out to be a good fit for the data (for mother version: χ2 /df = 1.57, RMSEA = 0.03, CFI = 0.99, TLI = 0.99, SRMR = 0.06, for father version: χ2 /df = 2.43, RMSEA = 0.06, CFI = 0.98, TLI = 0.97, SRMR = 0.08) but, it was not in line with our assumption of equivalence of concept items for both versions.

Finally, in the following confirmatory factor analysis, we tested the two-factor model with 52 items for both versions of BAPCQ. For the opposite versions (e.g., communication with the mother), we did not include questions that obtained factor loadings below 0.30 in one of them (e.g., communication with the father). Accordingly, we removed items 18 and 38 from the version of communication with the mother, and items 33, 52, and 57 from the version of communication with the father. The factor loadings for the items in the version of the questionnaire designed to assess maternal communication had values from 0.503 to 0.875 for the statement-action consistency scale and from 0.304 to 0.836 for the verbal-non-verbal consistency scale, whereas in the version for communication with the father, they had values from 0.580 to 0.887 for the statement-action consistency scale and from 0.334 to 0.830 for the verbal-non-verbal consistency scale.

We accepted the results of the third confirmatory factor analysis which confirmed a two-factor model with 52 items that were well-fitted to the data for both versions. We kept 26 items for the verbal-non-verbal consistency scale (16 items with reverse scoring) and 26 for the statement-action consistency scale (20 items with reverse scoring). Goodness of fit indices for the communication-with-mother version of the questionnaire were as follows: χ2 /df = 1.58, RMSEA = 0.03, CFI = 0.99, TLI = 0.99, SRMR = 0.06, and for the communication-with-father version: χ2 /df = 2.34, RMSEA = 0.05, CFI = 0.98, TLI = 0.98, SRMR = 0.07.

### 3.2. Internal Reliability

The result of Cronbach’s internal reliability for two scales of both versions of the questionnaire turned out to be satisfactory and was above 0.90. These results are shown in Table 5.

### 3.3. Validity of the BAPCQ

Pearson’s correlation coefficient was used to determine criterion validity. We obtained high (>0.5), moderate (>0.3) and low (<0.3) correlations between the BAPCQ scales and the scales of validation instruments. In the BAPCQ communication-with-mother version, high correlations were observed between Family Communication and Verbal-Non-verbal consistency (r = 0.715, *p* < 0.001) and Statement-action consistency scale (r = 0.591, *p* < 0.001), whereas in the communication-with-father version, we obtained moderate correlations in the same scales. The correlations between scales are presented in Table 6.

## 4. Discussion

Communication is an important resilience process that enables a family to function efficiently in times of crisis. One aspect of a clear communication process is consistency and unambiguity. Communication disruptions are normal for a family encountering difficulties. Błażek [10] and Walsh [23] emphasize that even in families that have functioned well up to a certain moment and are then put under severe stress, there may be disruptions in communicating with each other. The essence of communication understood in this way is the lack of fixed negative communication patterns that deepen family dysfunction. Referring to these assumptions the aim of our project was to consider the communication process, from the perspective of ambivalence perceived on two levels: words and actions, and the discrepancy between verbal and non-verbal communication. We decided to check how adult children brought up in families where both parents were involved in caring to perceive their communication with their mother and father. After reviewing the literature and the available methods regarding family communication (e.g., Ambivalence over Emotional Expression Questionnaire, Family Expressiveness Questionnaire), we noticed the lack in those areas of research and decided to create a questionnaire to assess the compliance-ambivalence of parental communication on two levels: compliance of words with deeds and verbal with non-verbal communication.

Confirmatory factor analysis confirmed that the two-factor model of the Błażek Ambivalent Parental Communication Questionnaire (BAPCQ) turned out to be well suited to the data both in the version of communication with the mother and with the father. We performed the confirmatory analysis three times in order to obtain the best measures of fit and to theoretically balance the questions in both questionnaires. Each version of the questionnaire consists of 52 items, 26 items for the verbal-non-verbal consistency scale and the same number of questions for the statement-action consistency scale. The scales of the questionnaires have a satisfactory reliability measured with Cronbach’s alpha, exceeding 0.9. 

We also confirmed the good criterion validity of BAPCQ with three other questionnaires measuring communication: FRAS-PL, WFRQ-PL, and SOR. The communication scales of the above-mentioned questionnaires correlated from low to high with the BAPCQ scales. The family communication scale from the Questionnaire of Family Assessment Scale [35] assesses aspects of general communication such as satisfaction with communication, showing feelings for one another, or showing respect in discussions. The family communication and problem scale in the Family Resilience Assessment Scale—PL [36] includes a multitude of communicative aspects. The participants not only assess the clarity of communication, but also openness and cooperation in solving problems, as well as learning from mistakes or coping with painful events. However, the communication process scale from the Walsh Family Resilience Questionnaire PL [44] examines three constructs such as: clarity of communication, open emotional expression, and cooperation in problem-solving. The described above scales obtained a satisfactory reliability and correctly assess communication aspects, therefore we used them for criterion validity. Nonetheless, only some of these items typically relate to the clarity and comprehensibility of the message. The WFRQ questionnaire tests clarity of communication by including questions such as: “We try to clarify information about our stressful situation and our options”, or “In our family, we are clear and consistent in what we say and do”. These items are relevant from the perspective of the family resilience communication process. Withal, we prepared a separate questionnaire for the inclusion of ambivalent communication, because we wanted to recognize the entire spectrum of inconsistent communication behavior. The BACPQ questionnaire helps reference the communication with the father and with the mother separately. Whereas, in each of the three questionnaires (WFRQ-PL, FRAS-PL, SOR), family communication was the unit of analysis.

Our study proved that the construct of cohesion-ambivalence of communication in the family psychology field of verbal-non-verbal behavior and statement-action is measurable and assessable. The questionnaire is intended for adults to retrospectively assess communication with parents, separately with the mother and the father. High scores indicate a high level of agreement and consistency in the assessed spheres, while low scores indicate highly ambivalent communication. High scores will be achieved by people whose one or both parents kept their word, were trustworthy, and behaved in accordance with their words. Low scores will be achieved by people whose parents broke their word, were not authentic, and did not fulfill their obligations. It should also be noted that the coherence of communication in the family, as well as cohesion, understood as family closeness, should be assessed not in relation to a single event, but to the entirety of family life.

The BACPQ extends insight into the clarity and consistency of communication between each parent and child separately. Long-term observations of family therapists and researchers of family resilience [23,32,45,46] confirm that confused communication, although it disturbs the functioning of the whole family, does not have to affect all family members, and is conditioned by specific relationships between individuals. Błażek [10] indicates that the earlier the ambivalence in communication between parents and children is detected, the earlier the opportunity to repair the relationship is gained. Families can be supported by therapists by working on acquiring appropriate communication skills. On the other hand, communication patterns are passed down from generation to generation, therefore interventions in adult children may turn out to be important, due to the fact that they acquired certain communication patterns in their families of origin, which they will then perpetuate in future relationships with partners and pass on to their children. In conclusion, the BACPQ questionnaire is a good method to measure consistency-ambivalence in parent-child communication and we recommend its use by scientists for their research, therapists for assessment and work with families experiencing communication difficulties.

As in most cases, our study also has limitations. One is that the majority of the study group were participants in early adulthood, mostly women. Secondly, we focused on the study of communication with parents only from the child’s perspective while not controlling for the perspective of mothers and fathers. Thirdly, the recruitment of participants via the Internet and the lack of control of socio-demographic variables may have created and implied a selection effect in favor of the more educated, higher social strata of the population.

Despite the limitations, we can indicate practical implications for the use of the BAPCQ questionnaire. The questionnaire allows for comparisons between countries in terms of the processes of communication with both parents. BAPCQ might be useful in scientific research and practical work with families of different structures and at different stages of family life, offering some insight into potential difficulties. Therapists using the BAPCQ questionnaire will be able to support both parents and protect children against the harm caused by communication inconsistency and therefore against emotional and relational difficulties in adulthood.

Our research on family communication is related to an important change in thinking about negative human experiences that affect one’s life, health, and relationships. As Narayan, et al. [47] point out, instead of focusing attention on negative consequences, we focus on prevention. Such thinking has important consequences not only for individuals (higher resilience, better health), but also for entire families and social groups (better relationships, stopping the intergenerational transmission of negative patterns). This line of research is connected with one of the five subtypes of childhood maltreatment- verbal/emotional [48]. Our study on the processes of communication will be continued and we would like to include more men, who were underrepresented in the present study, as well as participants in middle and late adulthood. We also plan to include family structures other than nuclear—for example, single-parent families, foster care, adoptions, same-sex families and so on. It would be interesting to use BAPCQ on family members from different generations to examine family communication patterns. 

## 5. Conclusions

Family resilience focuses on communication, which occurs in all family resilience processes. Our questionnaire helps explore this area in more detail and diagnose any potential difficulties in communication. The construction of BAPCQ was successful. The questionnaire consists of 52 items. We reserved 26 items for the verbal-non-verbal consistency scale and 26 for the statement-action consistency scale. The scales proved to have satisfactory reliability and criterion validity with FRAS-PL, WFRQ-PL, and SOR. Separate versions of the questionnaire for mothers and fathers make it possible to assess and detect differences in the child’s communication with both parents. BAPCQ reveals differences in two spheres of parents’ functioning in communication with a child: consistency of statements and actions, and consistency of verbal and non-verbal communication.

## Figures and Tables

**Table 1:** Subscales of questionnaires measuring family communication.

Questionnaire	Authors	Communication Scales
Family Resiliency Assessment Questionnaire	Sixbey, M. T.	Family Communication and Problem Solving
Walsh Family Resilience Questionnaire	Walsh, F.	Overarching scale: Communication and Problem-solving Processes Communication subscales: Clear, consistent messages Open Emotional Expression Collaborative Problem-solving
Family Assessment Scales	Margasiński, A.	Communication scale
Marriage Communication Questionnaire	Plopa, M.	Overarching scale: Assessment of the wife’s/partner’s behavior Assessment of husband’s/partner’s behavior Assessment of the wife’s/partner’s own behavior Assessment of the husband’s/partner’s own behavior Communication subscales: Support Commitment Depreciation
Family Relations Questionnaire	Plopa, M.; Połomski, P.	Overarching scale: My family My parents as a married couple My mother My father Me in my mother’s eyes—what my mother thinks of me Me in my father’s eyes—what my father thinks of me Communication subscales: Communication scale
Ambivalence over Emotional Expression Questionnaire	King, L. A.; Emmons, R. A.	Expressiveness, Social desirability, Intense ambivalence
Family Expressiveness Questionnaire	Halberstadt, A. G.	Affect dimension Power dimension (positive dominant, positive non-dominant, negative dominant, negative non-dominant)
Communication patterns questionnaire	Carvalhal, T.; Nazaré, B.	Positive Symmetrical Pattern, Negative Alternate Pattern, Negative Complementary Pattern.

**Table 2:** Characteristic of participants (*n* = 404).

Gender, % (*n*)	
Female	79.0 (319)
Male	21.0 (85)
Age, M ± SD	24.83 ± 7.87
Range of age	18–61
18–35, % (*n*)	90.6% (366)
35–63, % (*n*)	9.4% (38)
Level of education, % (*n*)	
Primary	1.0 (4)
Vocational	1.7 (7)
Secondary	69.1 (279)
Higher	28.2 (114)
Place of residence, % (*n*)	
The country	12.9 (52)
City up to 100,000 residents	16.1 (65)
City from 100,000 up to 500,000 residents	22.8 (92)
City over 500,000 residents	48.3 (195)

**Table 3:** Fit indexes of different models.

Model	χ2 /df	RMSEA	CFI	TLI	SRMR
Communication with mother					
with 62 items	2.12	0.05	0.97	0.97	0.07
with 54 items	1.57	0.03	0.99	0.99	0.06
with 52 items	1.58	0.03	0.99	0.99	0.06
Communication with father					
with 62 items	2.9	0.06	0.96	0.96	0.09
with 55 items	2.43	0.06	0.98	0.97	0.08
with 52 items	2.34	0.05	0.98	0.98	0.07

**Table 4:** Factor loadings for 62-item model.

	Communication with Mother			Communication with Father		
Item	Estimate	Std. Estimate	*p*	Estimate	Std. Estimate	*p*
Statement-action consistency scale						
1.	1.000	0.661	0.000	1.000	0.727	0.000
3.	1.328	0.792	0.000	1.142	0.790	0.000
6.	1.167	0.748	0.000	1.056	0.779	0.000
8.	1.262	0.723	0.000	1.131	0.794	0.000
10.	1.102	0.703	0.000	1.004	0.716	0.000
12.	1.404	0.795	0.000	1.136	0.780	0.000
15.	0.722	0.507	0.000	0.718	0.587	0.000
17.	1.181	0.639	0.000	0.920	0.664	0.000
20.	1.286	0.773	0.000	1.138	0.821	0.000
22.	1.273	0.716	0.000	1.094	0.783	0.000
25.	1.430	0.860	0.000	1.275	0.856	0.000
27.	1.380	0.810	0.000	1.215	0.849	0.000
30.	1.446	0.852	0.000	1.290	0.868	0.000
32.	1.517	0.874	0.000	1.156	0.827	0.000
35.	1.415	0.852	0.000	1.300	0.886	0.000
37.	1.353	0.740	0.000	1.090	0.763	0.000
40.	1.412	0.824	0.000	1.176	0.818	0.000
42.	1.413	0.755	0.000	1.091	0.760	0.000
45.	0.895	0.627	0.000	0.976	0.750	0.000
47.	1.391	0.827	0.000	1.223	0.858	0.000
49.	1.199	0.697	0.000	0.981	0.720	0.000
51.	1.431	0.810	0.000	1.148	0.856	0.000
54.	0.883	0.579	0.000	0.947	0.723	0.000
56.	0.987	0.583	0.000	0.832	0.651	0.000
57.	0.504	0.294	0.000	0.514	0.403	0.000
58.	1.358	0.751	0.000	1.184	0.810	0.000
60.	1.331	0.789	0.000	1.212	0.846	0.000
Verbal-non-verbal consistency scale						
2.	1.000	0.310	0.000	1.000	0.351	0.000
4.	1.878	0.347	0.000	1.191	0.365	0.000
5.	2.856	0.574	0.000	2.015	0.618	0.000
7.	1.584	0.367	0.000	1.220	0.415	0.000
9.	1.798	0.428	0.000	1.350	0.497	0.000
11.	3.258	0.631	0.000	1.591	0.497	0.000
13.	1.270	0.329	0.000	1.319	0.424	0.000
14.	2.596	0.487	0.000	1.703	0.539	0.000
16.	1.405	0.367	0.000	1.251	0.411	0.000
18.	1.916	0.360	0.000	0.677	0.206	0.001
19.	4.081	0.674	0.000	2.483	0.681	0.000
21.	3.431	0.686	0.000	2.244	0.694	0.000
23.	2.476	0.559	0.000	2.099	0.686	0.000
24.	2.433	0.483	0.000	1.665	0.541	0.000
26.	3.625	0.781	0.000	2.663	0.801	0.000
28.	3.405	0.750	0.000	2.564	0.823	0.000
29.	4.410	0.782	0.000	2.507	0.745	0.000
31.	2.875	0.524	0.000	1.705	0.513	0.000
33.	0.924	0.230	0.000	1.097	0.429	0.000
34.	3.588	0.678	0.000	1.887	0.605	0.000
36.	3.088	0.637	0.000	1.786	0.569	0.000
38.	1.871	0.352	0.000	0.727	0.212	0.004
39.	3.717	0.625	0.000	2.024	0.576	0.000
41.	0.668	0.176	0.000	0.324	0.120	0.023
43.	2.627	0.567	0.000	1.774	0.571	0.000
44.	3.596	0.720	0.000	1.907	0.615	0.000
46.	0.410	0.082	0.141	0.081	0.026	0.646
48.	0.741	0.132	0.036	0.617	0.187	0.005
50.	0.040	0.008	0.878	0.047	0.015	0.795
52.	0.833	0.201	0.001	0.923	0.332	0.000
53.	2.390	0.419	0.000	1.275	0.375	0.000
55.	1.645	0.344	0.000	1.041	0.342	0.000
59.	0.599	0.128	0.016	0.248	0.086	0.101
61.	4.272	0.833	0.000	2.695	0.827	0.000
62.	4.198	0.747	0.000	2.368	0.683	0.000

**Table 5:** Cronbach’s Alpha for scales of BAPCQ.

Scales	α
Communication with mother	
Statement-action consistency	0.97
Verbal-non-verbal consistency	0.93
Communication with father	
Statement-action consistency	0.98
Verbal-non-verbal consistency	0.93

**Table 6:** Correlations between the BAPCQ and scale of validation instruments.

Scales of BAPCQ	FCPS	CP	FC
Communication with mother			
Statement-action consistency	0.305 ***	0.255 ***	0.591 ***
Verbal-non-verbal consistency	0.354 ***	0.326 ***	0.715 ***
Communication with father			
Statement-action consistency	0.278 ***	0.190 **	0.370 **
Verbal-non-verbal consistency	0.279 ***	0.207 **	0.460 ***

Note. FCPS—family communication and problem-solving (FRAS-PL), CP—Communication Process (WFRQ-PL), FC—Family Communication (SOR). ** *p* < 0.01; *** *p* < 0.001.

## Data Availability

Not applicable.
